# GRP78‐mediated antioxidant response and ABC transporter activity confers chemoresistance to pancreatic cancer cells

**DOI:** 10.1002/1878-0261.12322

**Published:** 2018-08-07

**Authors:** Patricia Dauer, Nikita S. Sharma, Vineet K. Gupta, Alice Nomura, Vikas Dudeja, Ashok Saluja, Sulagna Banerjee

**Affiliations:** ^1^ Department of Pharmacology University of Minnesota Minneapolis MN USA; ^2^ Department of Surgery University of Miami FL USA; ^3^ Sylvester Comprehensive Cancer Center Miami FL USA

**Keywords:** chemoresistance, GRP78, Sp1, unfolded protein response

## Abstract

Chemoresistance is a major therapeutic challenge that plays a role in the poor statistical outcomes in pancreatic cancer. Unfolded protein response (UPR) is one of the homeostasis mechanisms in cancer cells that have been correlated with chemoresistance in a number of cancers including pancreatic cancer. In this study, we show that modulating glucose regulatory protein 78 (GRP78), the master regulator of the UPR, can have a profound effect on multiple pathways that mediate chemoresistance. Our study showed for the first time that silencing GRP78 can diminish efflux activity of ATP‐binding cassette (ABC) transporters, and it can decrease the antioxidant response resulting in an accumulation of reactive oxygen species (ROS). We also show that these effects can be mediated by the activity of specificity protein 1 (SP1), a transcription factor overexpressed in pancreatic cancer. Thus, inhibition of SP1 negatively affects the UPR, deregulates the antioxidant response of NRF2, as well as ABC transporter activity by inhibiting GRP78‐mediated ER homeostasis. Sp1 and NRF2 have been classified as nononcogene addiction genes and thus are imperative to understanding the molecular mechanism of resistance. These finding have huge clinical relevance as both Sp1 and GRP78 are overexpressed in pancreatic cancer patients and increased expression of these proteins is indicative of poor prognosis. Understanding how these proteins may regulate chemoresistance phenotype of this aggressive cancer may pave the way for development of efficacious therapy for this devastating disease.

**Abbreviations**
5‐FU: 5‐fluorouracil
ABC: ATP‐binding cassette
ARE: antioxidant response elements
ER: endoplasmic reticulum
GEM: gemcitabine
GRP78: glucose regulatory protein 78
KPC: LSLKras^G12D^, LSL‐Trp53^R172H^, Pdx‐1‐Cre
MDR: multidrug resistant
MTH: mithramycin
NS: nonsilencing siRNA
PDAC: pancreatic ductal adenocarcinoma
RLU: relative luciferase units
ROS: reactive oxygen species
SP1: specificity protein 1
UPR: unfolded protein response

## 1 Introduction

Pancreatic cancer is a devastating disease with relatively unchanged survival statistics for 70 years (ACS, 2017). In 2017, it is estimated that 53 670 people will be diagnosed with pancreatic cancer, and 43 090 people will die from the disease (ACS, 2017). FOLFIRINOX (oxaliplatin, irinotecan, leucovorin, and 5‐FU combination therapy) offers a slight survival benefit compared to gemcitabine alone, but only a small subset of patients can withstand the toxicity (Hessmann *et al*., 2017; Thierry Conroy *et al*., 2011). One of the main reasons for the poor survival statistics in this disease is due to drug resistance to these standards of care, such as gemcitabine, paclitaxel, and 5‐fluorouracil (5‐FU).

There are multiple mechanisms of chemoresistance employed by cancer cells. The unfolded protein response (UPR) contributes to chemoresistance, a homeostatic mechanism, which ameliorates stressful conditions to ensure cell survival. To survive the pressure created by the tumor microenvironment, including hypoxia and nutrient deprivation, tumor cells utilize the UPR pathways (Avril *et al*., 2017; Chevet *et al*., 2015; Lee, 2007; Ma and Hendershot, 2004). Multiple components of the UPR have been linked to advanced tumor stage and chemoresistance in cancer (Lee, 2014; Roller and Maddalo, 2013; Shen *et al*., 2017; Wang and Kaufman, 2014). For example, overexpression of PDIA5 in chronic myeloid leukemia shows increased resistance to imatinib (Chevet *et al*., 2015). Overexpression of XBP1s has been found in triple‐negative breast cancer, a notoriously resistant subset of breast cancer (Chevet *et al*., 2015; Fernandez *et al*., 2000). XBP1s also correlates with higher tumor grade, chemoresistance, and shorter survival in lymphoma (Wang and Kaufman, 2014). GRP78, the regulator of the UPR, has previously been correlated with poor patient prognosis in multiple cancers, including pancreatic cancer (Avril *et al*., 2017; Gifford *et al*., 2016; Ma and Hendershot, 2004; Niu *et al*., 2015; Wang and Kaufman, 2014). Based on correlation studies and direct overexpression leading to chemoresistance, numerous proteins in the UPR pathway have been targeted with small molecules in hopes to attenuate chemoresistance (Chevet *et al*., 2015; Gifford *et al*., 2016; Lee, 2014; Roller and Maddalo, 2013). In spite of a generous body of literature correlating chemoresistance to expression of genes involved in UPR, there have been almost no studies to elucidate the mechanism(s) by which UPR contributes to chemoresistance.

Upregulation of the oxidative stress response pathway is another mechanism of decreasing chemotherapy‐driven apoptosis. Management of oxidative stress decreases ROS accumulation in a cell, which in turn enhances detoxification and inhibits apoptotic cell death of tumor cells (Chio *et al*., 2016; DeNicola *et al*., 2011). The major transcription factor responsible for regulating oxidative stress response is nuclear factor erythroid 2‐like 2 (NRF2). NRF2 is downstream of the PKR‐like endoplasmic reticulum kinase sensor in the UPR and is one transcription factors that has been shown to regulate ABCC subfamily expression (Chartoumpekis *et al*., 2015). NRF2 normally acts like a tumor suppressor, but overactivation of its antioxidant response elements (ARE) activity leads to cell survival of normal and malignant cells (Chartoumpekis *et al*., 2015; DeNicola *et al*., 2011; Menegon *et al*., 2016). It has previously been reported that gemcitabine upregulates the NRF2 pathway, helping to mediate resistance (Avril *et al*., 2017; Palam *et al*., 2015).

Another well‐known mechanism of chemoresistance is through the ABC transporter family. ABC transporters drive the efflux of xenobiotics, including chemotherapeutics. High transporter activity keeps cellular chemotherapeutic compounds low, thereby decreasing their effective concentration within the cell. The most common transporters involved in chemoresistance are BRCP (ABCG2), MDR1 (ABCB1), and MRPs (ABCCs) (Choudhuri and Klaassen, 2006; Krishna and Mayer, 2000; Molnár and Zalatnai, 2007; Pang *et al*., 2014; Zhao *et al*., 2016).

A previously published study from our laboratory showed higher GRP78 expression in tumor ducts compared to nontumor tissue (Mujumdar *et al*., 2014). It was recently reported that GRP78 correlates with poor prognosis and chemoresistance in pancreatic cancer (Gifford *et al*., 2016; Niu *et al*., 2015). Further, it was shown that gemcitabine‐resistant cell lines have higher GRP78 expression compared to gemcitabine‐sensitive cell lines (Gifford *et al*., 2016). It has also been reported that patients with pancreatic cancer have an upregulation of ABC transporters, particularly ABCC3 and ABCC5 (Konig *et al*., 2005; Pang *et al*., 2014). However, it is unclear whether the ABC expression regulation correlates with PDAC tumor stages (Konig *et al*., 2005; Pang *et al*., 2014).

Specificity protein 1 (SP1) regulates many biological functions, including cell growth, differentiation, and survival, as well as tumor progression and metastasis (Banerjee *et al*., 2013; Beishline and Azizkhan‐Clifford, 2015; Deniaud *et al*., 2009; Hedrick *et al*., 2016; Kanai *et al*., 2006). Multiple studies have underscored the importance of SP1 in malignant tissues (Banerjee *et al*., 2013; Deniaud *et al*., 2009; Kanai *et al*., 2006). Interestingly, it has been reported in colon, gastric, pancreatic, and breast cancers that SP1 is overexpressed, whereas minimal‐to‐no SP1 expression is detected in normal differentiated cells (Banerjee *et al*., 2013; Deniaud *et al*., 2009; Kanai *et al*., 2006). We have recently published that upon downregulation of SP1, transcriptional regulation of GRP78 is adversely affected and cannot recover from chronic ER stress, which leads to cell death (Dauer *et al*., 2017).

In this manuscript, we show for the first time that modulation of GRP78 expression by either overexpressing or silencing has a profound effect on a number of independent pathways that are responsible for chemoresistance. We further show that the effect of GRP78 modulation is mediated by the activity of SP1, a transcription factor that plays a significant role in maintaining ER homeostasis. Thus, the inhibition of SP1 deregulates the antioxidant response of NRF2 as well as ABC transporter activity by inhibiting GRP78‐mediated ER homeostasis via the unfolded protein response.

## 2 Materials and methods

### 2.1 Cell culture and treatment

MIA PaCa‐2 (obtained from ATCC) was cultured in DMEM; high glucose, supplemented with 10% FBS and 100 units·mL^−1^ penicillin and 100 μg·mL^−1^ streptomycin. S2‐VP10 (a gift from Dr. Masato Yamamoto, University of Minnesota) and SU.86.86 (ATCC) were grown and propagated in RPMI, supplemented with 10% FBS, 100 units·mL^−1^ penicillin, and 100 μg·mL^−1^ streptomycin. All cells were maintained at 37 °C in a humidified air atmosphere with 5% CO_2_.

ON‐TARGETplus SMARTpool (pool of 4 siRNA) human SP1 (Dharmacon, Cat # L‐026959‐00‐0020) and GRP78 siRNA (Dharmacon, Cat # L‐008198‐00‐0020) were used for silencing experiments. Transfections were completed using DharmaFECT (Dharmacon) according to the manufacturer's instructions. Evidence of silencing GRP78 and SP1 is provided in Fig. S6.

Human HSPA5 siRNA (SMARTpool) Target Sequences: GCGCAUUGAUACUAGAAA; GAACCAUCCCGUGGCAUAA; GAAAGAAGGUUACCCAUGC; AGAUGAAGCUGUAGCGUAU
Human ON‐TARGETplus Nontargeting siRNA #2 Target Sequence: UGGUUUACAUGUUGUGUGA
Human SP1 siRNA (SMARTpool) Target Sequences: GCCAAUAGCUACUCAACUA; GAAGGGAGGCCCAGGUGUA; GGGCAGACCUUUACAACUC; CUACAGAGGCACAAACGUA

### 2.2 KPC tumor analyses

LSLKrasG12D; LSL‐Trp53R172H; and Pdx‐1‐Cre (KPC) mice of various ages (1–9 months) were euthanized, and pancreata were analyzed for mRNA and protein expression of GRP78, NRF2, and ABC transporters. Mice were sorted according to age and tumor status into nontumor and full‐tumor groups; that is, the 3‐month KPC mice with tumors were placed in the tumor group (ranging from 3 to 9 months).

### 2.3 Gene expression analyses

RT‐PCR: RNA was isolated from the cells according to the manufacturer's instructions using Trizol (Invitrogen; Carlsbad, CA, USA). Total RNA (2 μg) was used to make cDNA and perform real‐time PCR using the QuantiTect SyBr Green PCR Kit (Qiagen; Hilden, Germany) according to the manufacturer's instructions using Roche 480 real‐time PCR system. All data were normalized to the housekeeping gene 18S (Qiagen, Cat # QT00199367). Quantitative RT‐PCR primers for SP1 (Qiagen, Cat # QT01870449) and HSPA5 (GRP78) (Qiagen, Cat # QT 00096404).

### 2.4 ELISA

KPC or human pancreatic cancer patient serum was used to analyze the GRP78 serum levels, using the Enzo ELISA Kit, per manufacturer's instructions. GRP78 antibody is immobilized in a precoated plate. 100 μL of serum was pipetted in each well of a 96‐well plate in duplicate and allowed to bind overnight at 4 °C. The following morning, the serum was removed and fresh GRP78 standards were prepared and pipetted in duplicate. 100 μL of biotin‐conjugated GRP78 antibody was added to each well, followed by 100 μL HRP‐avidin secondary. Color formation was achieved using a TMB substrate for 30 min and was detected at 450 nm using a microplate reader.

### 2.5 Immunofluorescence

Paraffin‐embedded KPC mouse tissues were deparaffinized in xylene and then rehydrated in graded ethanol. After citrate antigen retrieval, tissues were blocked with Dako protein block and incubated with primary (GRP78, Abcam ab21685) at 1 : 1000 dilution. Slides were washed with PBS and incubated with Alexa Fluor 488 anti‐rabbit secondary antibody for 1 h. Slides were washed with PBS and mounted with prolong gold with DAPI (Invitrogen).

MIA PaCa‐2 and S2‐VP10 cells were plated in chamber slides and incubated for 1–24 h at 37 °C. The slides were treated with 400 nm (MIA PaCa‐2) or 100 nm (S2‐VP10) gemcitabine; fixed with 2% paraformaldehyde. The slides were incubated with 1 : 1000 dilution of rabbit polyclonal anticleaved caspase 3 antibody (Cell Signaling; Danvers, MA, USA) and a 1 : 1000 dilution of Alexa 488‐conjugated donkey anti‐rabbit IgG (Molecular Probes; Eugene, OR, USA). The slides were mounted using Prolong Gold antifade with 4′,6‐diamidino‐2‐phenylindole (Molecular Probes). Immunofluorescence images were obtained on a Leica DM6B with a 20× objective. Cleaved caspase was quantified using Image J software.

### 2.6 Immunohistochemistry

Paraffin‐embedded KPC mouse and human pancreatic cancer patient tissues were deparaffinized in xylene and then rehydrated in graded ethanol. Slides were steamed with pH 9 antigen retrieval (Dako). Endogenous peroxidases were blocked with a 3% hydrogen peroxide solution. Tissues were then blocked with Dako protein block and incubated with primary antibody overnight. Slides were stained with anti‐SP1 (Cell Signaling, Cat # 9389) and anti‐GRP78 (Cell Signaling, Cat # 3177) at 1 : 200 dilutions. Slides were washed with PBS, incubated with secondary anti‐rabbit antibody, and conjugated to horseradish peroxidase, for 30 min. Slides were washed again with PBS. Diaminobenzidine Peroxidase Substrate Kit (Vector Laboratories) was then added to slides. Primary antibody was omitted for negative controls. Slides were mounted with permount. Images were obtained on a Leica DM6B with a 20× objective.

### 2.7 Viability

MIA PaCa‐2, S2‐VP10, and SU.86.86 cells were seeded in a 96‐well plate (7000 cells/well) and allowed to adhere for 24 h. Cells were transfected with 20 nm nonsilencing siRNA (NS), siGRP78, or siSP1. Drug treatments used 400 nm (MIA PaCa‐2) or 100 nm (S2‐VP10) gemcitabine, 50 nm paclitaxel, or 5 μm 5‐FU, unless otherwise specified. Cell viability assays following siRNA or pharmacological inhibitors were performed using a WST‐8‐based cell cytotoxicity assay per the manufacturer's protocol (Dojindo; Rockville, MD, USA) and expressed after normalizing to untreated cells.

### 2.8 Dye Efflux Assay

MIA PaCa‐2 cells were plated in a 6‐well plate. NS, siGRP78, and siSP1 were transfected in two wells each and allowed to incubate for 15 h. Cells were then treated with 400 nm gemcitabine or 50 nm paclitaxel for 8 h. Cells were then gently scraped and were transferred equally into two flow cytometry tubes. In one set, NucBlue live dye was added and incubated on ice. In the second set of tubes, NucBlue live dye was added, along with 100 μm verapamil, and incubated at 37 °C for 30 min, and subsequently moved to ice. Flow cytometry was performed using the Pacific Blue and AmCyan. The percent of inhibition was calculated by (+ Verapamil) – (− Verapamil).

### 2.9 ATP Determination

MIA PaCa‐2 and S2‐VP10 cells were seeded in a 6‐well plate. NS, siGRP78, and siSP1 were transfected in two wells each and allowed to incubate for 15 h. Cells were then treated with 400 nm (MIA PaCa‐2) or 100 nm (S2‐VP10) gemcitabine for 24 h. Total cellular ATP was measured using the ENLITEN ATP assay system (Promega; Madison, WI, USA). Briefly, 1 μL trichloroacetic acid was added to 100 μL cell lysate to extract the ATP. Each sample was then neutralized with 900uL TAE (pH 8.0). A standard curve ranging from 10^−7^ m to 10^−11^ m was prepared using the provided 10^−7^ m ATP standard. Luminescence was recorded for each sample and standard.

### 2.10 ATPase Activity Assay

MIA PaCa‐2 and S2‐VP10 cells were seeded in a 6‐well plate. NS, siGRP78, and siSP1 were transfected in two wells each and allowed to incubate for 15 h. Cells were then treated with 400 nm (MIA PaCa‐2) or 100 nm (S2‐VP10) gemcitabine for 24 h. Cells were lysed in lysis buffer and analyzed for ATPase per manufacturer's instructions (Sigma; St. Louis, MO, USA). After assay incubation, plate was read at 620 nm for standards, blanks, and unknowns on a spectrophotometer.

### 2.11 ARE Reporter Assay

MIA PaCa‐2 and S2‐VP10 cells were seeded in a 24‐well plate. NS, siGRP78, and siSP1 were transfected in two wells each and allowed to incubate for 15 h. Cells were then transfected with the Cignal Reporter plasmids for ARE (Qiagen) and treated with 400 nm (MIA PaCa‐2) or 100 nm (S2‐VP10) gemcitabine for 24 h. Wells were washed with PBS, and 100 μL of passive lysis buffer was added per well. After 15 min of rocking in passive lysis buffer, plates were stored at −80 °C until ready to read. The dual luciferase kit (Promega) was used to measure activity using a luminometer. Each sample was treated in duplicate for each plasmid (duplicates for the negative reporter and duplicates for the ARE reporter).

### 2.12 Reactive Oxygen Species assay

MIA PaCa‐2 and S2‐VP10 cells were seeded in black, clear bottom 96‐well plates. NS, siGRP78, and siSP1 were transfected in two wells each and allowed to incubate for 15 h. Cells were then treated with 400 nm (MIA PaCa‐2) or 100 nm (S2‐VP10) gemcitabine, 50 nm paclitaxel, and 5 uM 5‐FU for 24 h. Media were removed and replaced with 5 uM H2DCFDA/phenol‐free media for 1 h at 37 °C. Cells were washed with PBS, and phenol‐free media was replaced. ROS was measured at 5‐min intervals for 1 h, with 492/517 ex/em filters. Results were expressed as ROS fluorescence per viability using a WST‐8 cell cytotoxicity assay (Dojindo).

### 2.13 *In vivo* study

8‐week‐old athymic nude mice were injected subcutaneously (right flank) with 10^6^ MIA PaCa‐2 cells suspended in Matrigel (Corning). Mice were randomized when the average tumor size reached 135 mm^3^. Mice were randomized into the following groups: 1. saline; 2. mithramycin 0.3 mg·kg^−1^; 3. mithramycin 0.6 mg·kg^−1^; 4. gemcitabine 50 mg·kg^−1^; 5. mithramycin 0.3 mg·kg^−1^ + gemcitabine 50 mg·kg^−1^; and 6. mithramycin 0.6 mg·kg^−1^ + gemcitabine 50 mg·kg^−1^. Saline and all mithramycin groups had seven mice each; gemcitabine had five mice. Mithramycin was administered intraperitoneally three times per week. Gemcitabine was administered intraperitoneally two times per week. Tumors were measured weekly with a digital calipers. Mice were euthanized when tumors reached 900 mm^3^. All procedures were approved by the University of Minnesota Institutional Animal Care and Use Committee (IACUC).

### 2.14 Statistical analysis

Values are expressed as the mean ± SEM. All *in vitro* experiments were performed at least three times. The significance between any two samples was analyzed by t‐test, and values of *P* < 0.05 were considered statistically significant.

*Analyses containing human specimens: The experiments were undertaken with the understanding and written consent of each subject. The study methodologies conformed to the standards set by the Declaration of Helsinki. The study methodologies were approved by University of Minnesota IRB.

## 3 Results

### 3.1 GRP78 expression correlates with tumor progression

**Figure 1:**
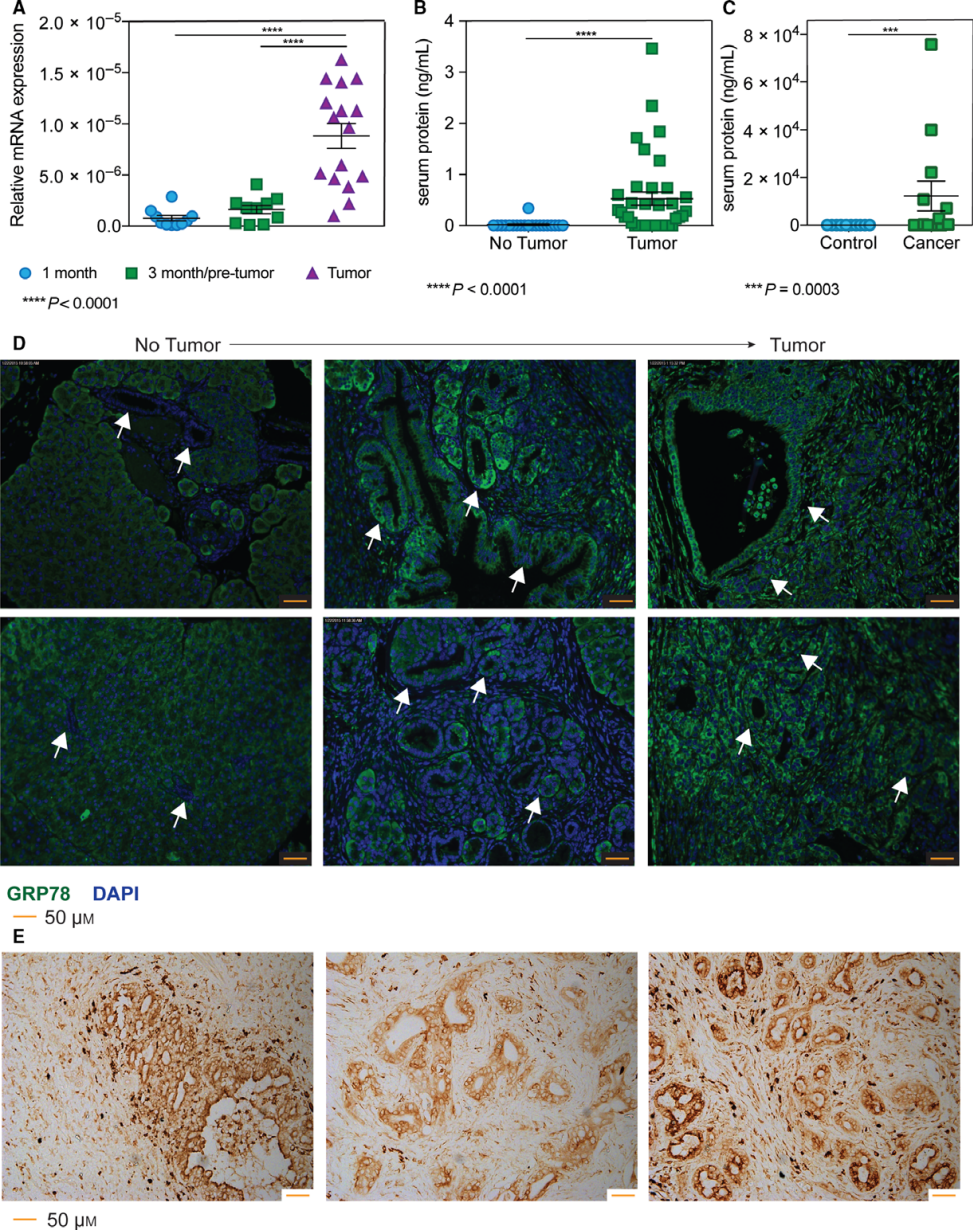
GRP78 expression correlates with tumor progression. Analysis using KPC pancreata ranging from 1 to 9 months found that (A) GRP78 mRNA expression is overexpressed in pancreata with tumor compared to 1‐ or 3‐month‐old KPC mice, and (B) GRP78 in serum from mice with tumors compared to mice without tumors was significantly greater. Serum from human patients also had elevated GRP78 compared to healthy controls (C). GRP78 protein expression (FITC) was found to be overexpressed in the ductal/pseudoductal regions in (D) the tumor‐bearing mice compared to mice without tumors (highlighted by arrows), as well as (E) human patient biopsy slides. Images were acquired at 20× magnification.

We found significantly increased GRP78 mRNA expression in tumor‐bearing mice (8.82e^−6^) compared to 1‐month (7.76e^−7^) and 3‐month mice (1.62e^−6^) (Fig. 1A). Serum protein level of GRP78 was also significantly increased (0.53 ng·mL^−1^) compared to the nontumor‐bearing controls (0.02 ng·mL^−1^) (Fig. 1B). Further, we analyzed human patient serum for levels of GRP78, and found a similar increase in GRP78 expression (12 258 ng·mL^−1^) compared to healthy controls (5.25 ng·mL^−1^) (Fig. 1C). Consistent with our mRNA data, we looked at GRP78 protein expression in the KPC pancreata by immunofluorescence staining and found an increase in the ductal expression of GRP78 in the tumor‐bearing group compared to the nontumor group (Fig. 1D). Additionally, we found GRP78 to be overexpressed in the ducts/pseudoducts of patient biopsy punches, which is consistent with recently published studies (Fig. 1E) (Gifford *et al*., 2016; Niu *et al*., 2015).

### 3.2 Silencing GRP78 combined with chemotherapeutics increases cell death

**Figure 2:**
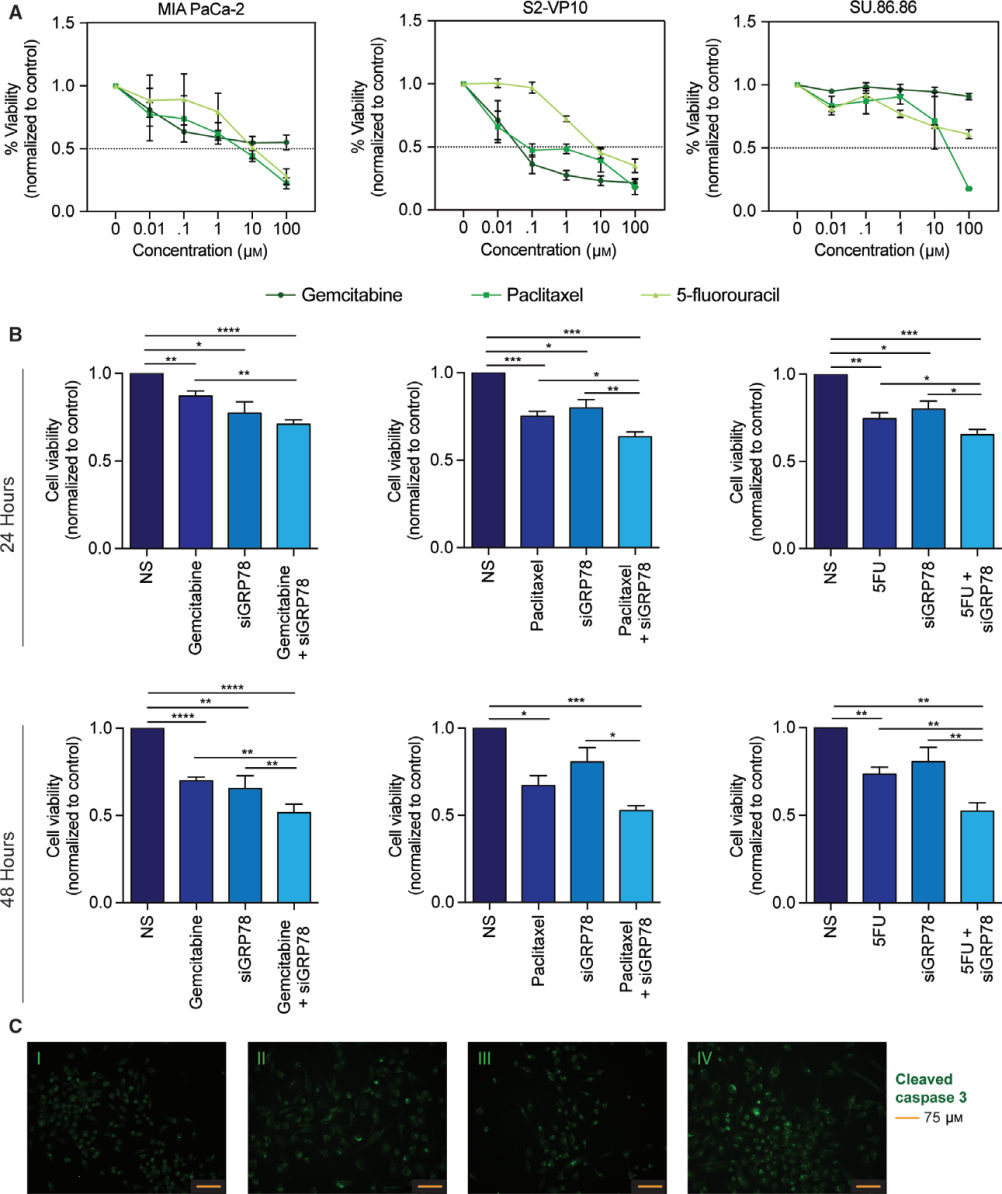
Silencing GRP78 combined with chemotherapeutics increases cell death. MIA PaCa‐2, SU.86.86, and S2‐VP10 cells were all treated with a dose response (0–100 μm) of gemcitabine, paclitaxel, and 5‐fluorouracil for 48 h (A). MIA PaCa‐2 cells transfected with siGRP78 and treated with 400 nm gemcitabine, 50 nm paclitaxel, or 5 μm 5‐fluorouracil for 24 and 48 h resulted in decreased viability compared to treatment or silencing alone (B). MIA PaCa‐2 cells transfected with nonsilencing (NS) siRNA (C‐I), 400 nm gemcitabine (C‐II), siGRP78 (C‐III), or siGRP78 +  gemcitabine (C‐IV) for 24 h. Cells were fixed and probed with a cleaved caspase 3 antibody for apoptosis, and images were acquired at 20× magnification.

Three pancreatic cancer cell lines: MIA PaCa‐2, SU.86.86, and S2‐VP10 were used to demonstrate the chemoresistance to relevant PDAC drugs (gemcitabine, paclitaxel, and 5‐fluorouracil). Figure 2A is a dose response curve for each cell line with the three drugs after 48 h of treatment. To determine whether silencing GRP78 would diminish chemoresistance in the same cell lines, cells were transfected with siGRP78 and treated with gemcitabine, paclitaxel, or 5‐FU for 24–48 h. We found that combining chemotherapeutics (using greater than IC50 concentrations) with siGRP78 resulted in more cell death than silencing or drug treatment alone in 24 and 48 h (Fig. 2B, Fig. S1A,B). In addition to cell viability, apoptosis was detected using immunofluorescence by probing with a cleaved caspase 3 antibody. MIA PaCa‐2 (Fig. 2C, Fig. S2A) and S2‐VP10 (Fig. S2B) were transfected with nonsilencing (NS) siRNA, NS + gemcitabine, siGRP78, or siGRP78 +  gemcitabine for 24 h. Our results show increased cleaved caspase 3 in siGRP78 +  gemcitabine compared to gemcitabine or NS alone.

### 3.3 Silencing GRP78 combined with chemotherapeutic compounds decreases ABC transporter activity in pancreatic cancer cells

**Figure 3:**
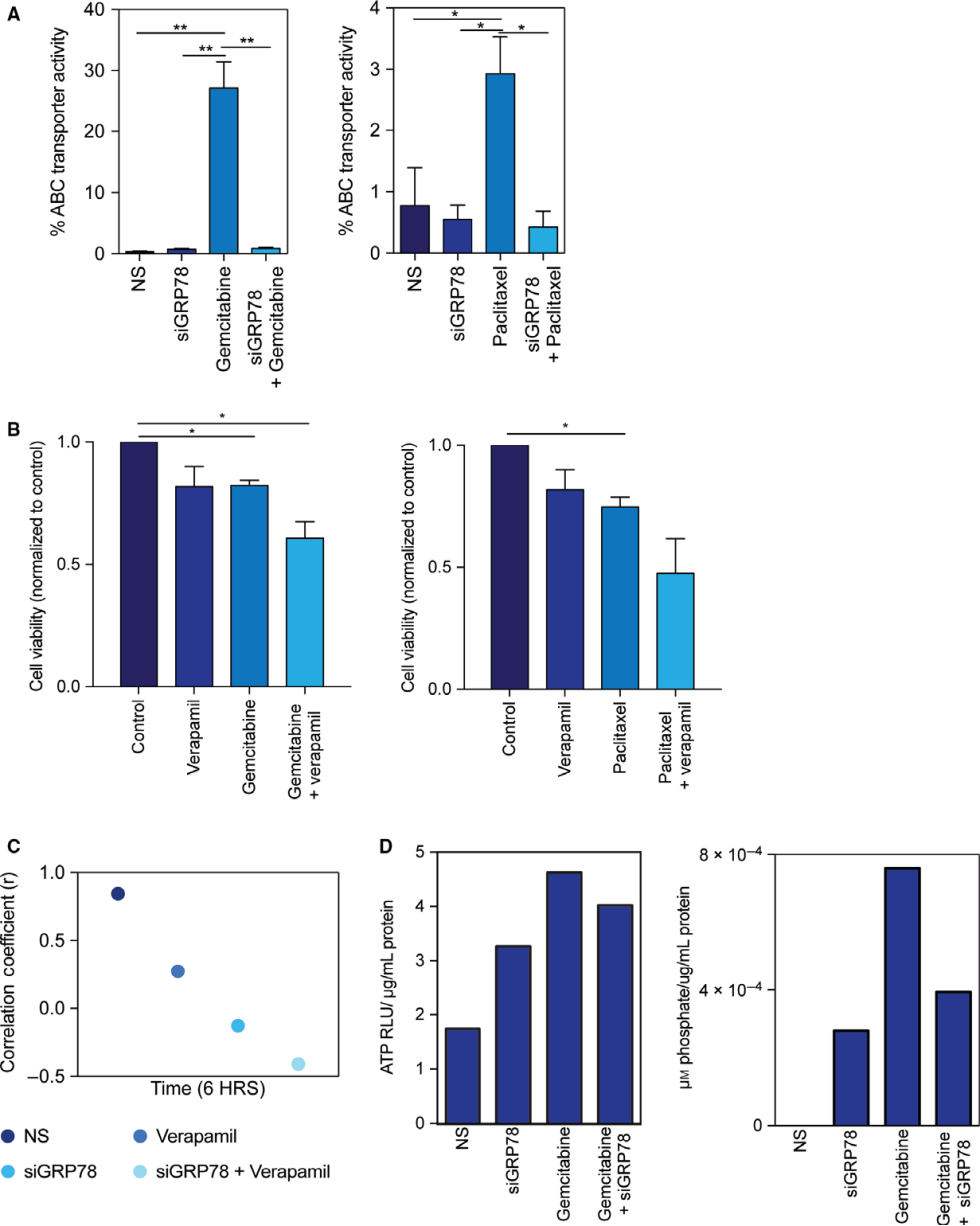
Silencing GRP78 combined with chemotherapeutic compounds decreases ABC transporter activity in pancreatic cancer cells. MIA PaCa‐2 cells transfected with siGRP78 and treated with 400 nm gemcitabine or 50 nm paclitaxel for 8 h ± verapamil, and analyzed by flow cytometry (A). MIA PaCa‐2 cells were treated with verapamil + chemotherapeutics for 24 h to determine cell viability (B). Pearson's coefficient was calculated for ER tracker and calcium co‐localization in MIA PaCa‐2 cells transfected with siGRP78 and treated with 100 μm verapamil for 6 h (C). MIA PaCa‐2 cells transfected with siGRP78 and treated with 400 nm gemcitabine for 24 h to measure total ATP (D) and ATPase (E). Figures are representative of three separate experiments.

ATP‐binding cassette transporters are a known mechanism of chemoresistance. Our results showed numerous ABC transporters to be overexpressed in KPC tumors compared to 1‐month KPC without tumors (Table 1). Further, using a dye efflux assay, we found that gemcitabine and paclitaxel alone both increased the % efflux of cells (27.1% and 2.9%, respectively). Conversely, nonsilencing (0.35–0.77%) and siGRP78 (0.43–0.88%) had minimal efflux activity. When MIA PaCa‐2 cells were silenced with siGRP78 and treated with gemcitabine, the % efflux decreased back to baseline (0.68%). siGRP78 and paclitaxel had a similar trend as gemcitabine, and the combination decreased the % efflux to 0.56% (Fig. 3A).

**Table 1:** mRNA expression of ABC transporter genes during pancreatic cancer progression in a KPC mouse

	1 month	SEM	3 months/pretumor	SEM	Tumor	SEM
ABCA1	6.011E‐07	2.69E‐07	1.117E‐06**	7.82E‐07	1.125E‐05**	1.41E‐06
ABCB1	4.539E‐08	2.05E‐08	3.096E‐08**	1.63E‐08	2.172E‐06**	7.95E‐07
ABCC1	6.210E‐07	3.60E‐07	6.897E‐07**	3.58E‐07	5.478E‐06**	1.25E‐06
ABCC2	4.731E‐06	3.32E‐06	3.076E‐05	2.46E‐05	5.852E‐05	4.22E‐05
ABCC4	1.050E‐07	5.19E‐08	1.302E‐07**	7.25E‐08	2.941E‐06**	7.21E‐07
ABCC5	8.264E‐07	3.34E‐07	1.167E‐06*	4.71E‐07	4.990E‐06*	7.94E‐07
ABCG2	2.247E‐07	6.90E‐08	1.008E‐05	7.53E‐06	2.493E‐06	1.29E‐06

*< 0.001; **< 0.0001.

The dye efflux assay utilizes verapamil to effectively block transport, but also as a calcium channel blocker, blocks calcium channels. We next wanted to determine the effect on viability and calcium changes when blocking transporters using verapamil with and without siGRP78 or drugs. The combination of verapamil and drugs in MIA PaCa‐2 cells (Fig. 3B, Fig. S3A) and S2‐VP10 cells (Fig. S3B) decreased cell viability in 24 h more than verapamil or drug alone. Next, we transfected MIA PaCa‐2 cells with NS, siGRP78, NS + verapamil, or siGRP78 +  verapamil for 1 and 6 h, and stained cells with ER tracker and Fluo4 (calcium dye). Pearson's coefficients were calculated for each group. After 1 h, *r* ranged from 0.3166 to 0.7254 (data not shown). However, in 6 h, NS had an *r* value of 0.8848; siGRP78 *r* = −0.1272; verapamil *r* = −0.5227; and siGRP78 +  verapamil *r* = −0.4092 (Fig. 3C), indicating a negative correlation between calcium and ER tracker.

ATP‐binding cassette transporters are fueled by ATP. To determine how GRP78 (an ER resident protein) effectively decreases the ABC transporter activity, we silenced GRP78 and measured the total ATP and ATPase per condition described in MIA PaCa‐2 cells. Results are expressed as relative luciferase units (RLU) per μg/mL protein. Gemcitabine alone increased ATP (4.62) as compared to nonsilenced (1.74) and siGRP78 (3.26). Gemcitabine with siGRP78 decreased the total ATP (4.02) compared to gemcitabine alone (Fig. 3D). The amount of ATPase corresponds with the total cellular ATP. ATPase is expressed as μm phosphate per μg/mL protein. Gemcitabine had an increase in the ATPase (7.59e^−4^) compared to NS (0) or siGRP78 (2.80e^−4^) (Fig. 3E), indicating that more ATP is being utilized. Gemcitabine with siGRP78 decreased the amount of ATPase compared to gemcitabine alone (3.94), indicating that less ATP is being utilized with the combination.

### 3.4 Silencing GRP78 combined with chemotherapeutic compounds decreases antioxidant response in activity in pancreatic cancer cells

**Figure 4:**
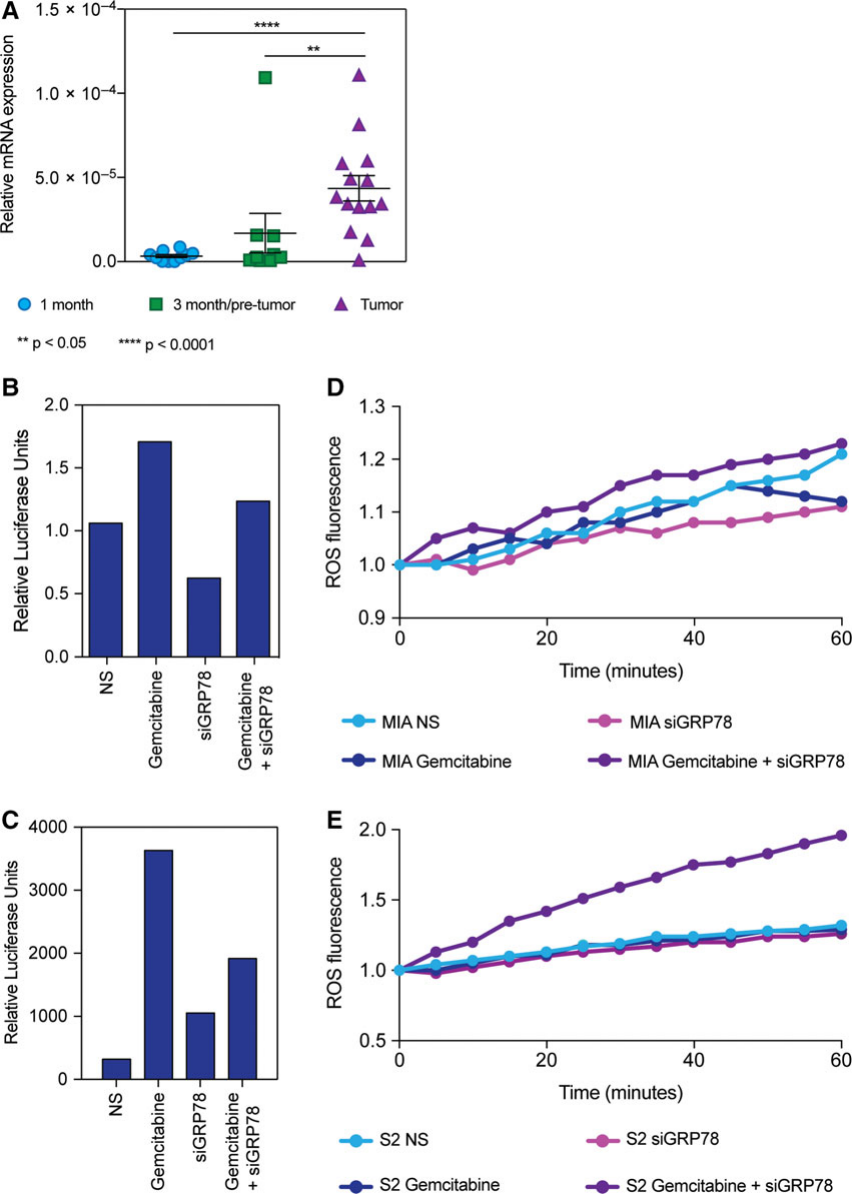
Silencing GRP78 combined with chemotherapeutic compounds decreases antioxidant response in activity in pancreatic cancer cells. Analysis using KPC pancreata ranging from 1 to 9 months found that (A) NRF2 mRNA expression is overexpressed in pancreata with tumor compared to 1‐ or 3‐month‐old KPC mice. MIA PaCa‐2 cells transfected with siGRP78 and treated with 400 nm gemcitabine for 24 h to measure ARE activity (B). S2‐VP10 cells transfected with siGRP78 and treated with 100 nm gemcitabine for 24 h to measure ARE activity (C). ROS measurement of MIA PaCa‐2 (D) and S2‐VP10 (E) cells after 24 h of treating with gemcitabine and siGRP78.

NRF2 is also an important mechanism of chemoresistance, by binding to the antioxidant response elements and transcribing detoxification genes, as well as some ABC transporters. We found that NRF2 mRNA expression is overexpressed in full‐tumor KPC pancreata (4.35e^−5^) compared to 1‐month (3.33e^−6^) and 3‐month pancreata without tumors (1.69e^−5^) (Fig. 4A). In terms of activity, gemcitabine modestly increased the NRF2 activity in MIA PaCa‐2 cells (1.71 RLU), as measured by a luciferase reporter of the antioxidant response elements (representative results shown). siGRP78 modestly decreased NRF2 activity (0.62 RLU), and when combined with gemcitabine (1.23 RLU), it decreases the activity closer to nonsilenced cells (NS) (1.06 RLU) (Fig. 4B). S2‐VP10 cells had a similar trend, but resulted in much more NRF2 activity. Gemcitabine increased the NRF2 activity (3632 RLU), and siGRP78 was slightly higher than NS (1055 RLU), and when combined with gemcitabine (1915 RLU), it decreases the activity closer to NS (320 RLU) (Fig. 4C). Further, we observed that the total ROS detected with DCFDA dye was increased in the siGRP78 + gemcitabine compared to NS, siGRP78, or treatment alone in MIA PaCa‐2 (Fig. 4D) as well as in S2‐VP10 (Fig. 4E), indicating that the UPR‐NRF2/ARE signaling is not able to decrease the amount of ROS being produced in the combination treatment.

### 3.5 SP1 is required for ER homeostasis and affects chemoresistance in pancreatic cancer cells, similar to GRP78

**Figure 5:**
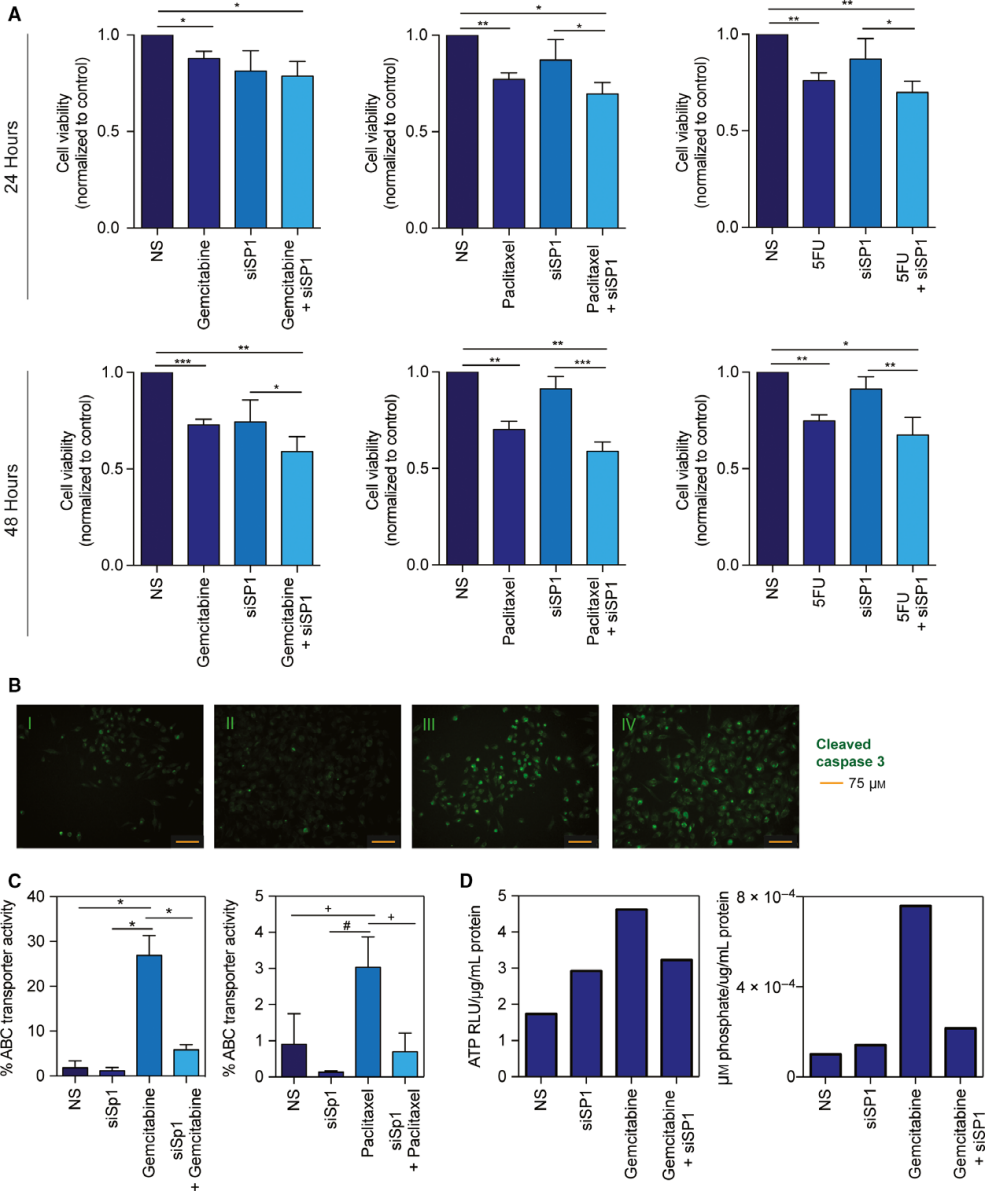
SP1 is required for ER homeostasis and affects chemoresistance in pancreatic cancer cells, similar to GRP78. MIA PaCa‐2 cells transfected with siSP1 and treated with 400 nm gemcitabine, 50 nm paclitaxel, or 5 μm 5‐fluorouracil for 24 and 48 h resulted in decreased viability compared to treatment or silencing alone (A). MIA PaCa‐2 cells transfected with nonsilencing (NS) siRNA (B‐I), 400 nm gemcitabine (B‐II), siSP1 (B‐III), or siSP1 +  gemcitabine (B‐IV) for 24 h, and probed with a cleaved caspase 3 antibody for apoptosis. Images were acquired at 20× magnification. MIA PaCa‐2 cells transfected with siSP1 and treated with 400 nm gemcitabine or 50 nm paclitaxel for 8 h ± verapamil, and analyzed by flow cytometry (C). MIA PaCa‐2 cells transfected with siSP1 and treated with 400 nm gemcitabine for 24 h to measure total ATP (D) and ATPase (E).

We have previously shown that SP1 is required for ER homeostasis in pancreatic cancer (Dauer *et al*., 2017). MIA PaCa‐2 (Fig. 5A) and S2‐VP10 cells (Fig. S4A) were treated with NS siRNA, NS + gemcitabine, siSP1, or siSP1 +  gemcitabine for 24–48 h. We observed similar chemosensitivity as with siGRP78 when comparing cell viability with silencing SP1. In addition to cell viability, apoptosis of cells untreated vs. treated was detected using immunofluorescence by probing with a cleaved caspase 3 antibody. Again, we observed similar results as with silencing GRP78 and found that combining siSP1 with drugs resulted in more cell death (Fig. 5B, Fig. S4B,C).

Using a dye efflux assay, we found that gemcitabine and paclitaxel alone both increased the % efflux of MIA PaCa‐2 cells (26.9% and 3.0%, respectively). Conversely, nonsilencing (0.90–1.85%) and siSP1 (0.13–1.18%) had minimal efflux activity. The combination of siSP1 and gemcitabine decreased the % efflux toward baseline (5.83%), whereas siSP1 and paclitaxel modestly decreased efflux (0.70%) (Fig. 5C). We also determined the total ATP and ATPase per condition in MIA PaCa‐2 cells. Gemcitabine alone increased the ATP (4.62) compared to nonsilenced (1.72) and siSP1 (2.92) (Fig. 5D). Gemcitabine with siSP1 decreased the total ATP compared to gemcitabine alone (3.23). The amount of ATPase corresponds with the total cellular ATP, in that gemcitabine had an increase in the ATPase (7.59e^−4^), indicating that more ATP is being utilized compared to NS (0) or siSP1 (1.41e^−4^) (Fig. 5E). Gemcitabine with siSP1 decreased the amount of ATPase compared to gemcitabine alone (2.15e^−4^), indicating that less ATP is being utilized.

### 3.6 Inhibition of SP1 *in vivo* overcomes gemcitabine‐induced chemoresistance

**Figure 6:**
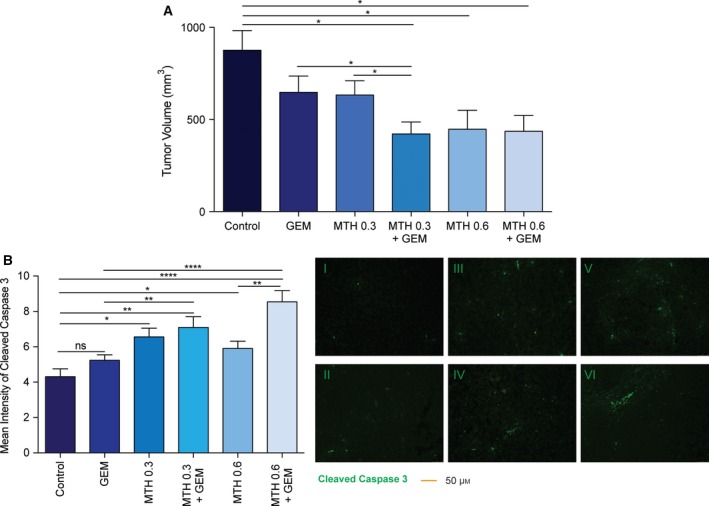
Inhibition of SP1 *in vivo* overcomes gemcitabine‐induced chemoresistance. MIA PaCa‐2 cells injected subcutaneously into athymic nude mice and treated with gemcitabine (50 mg·kg^−1^ twice weekly), mithramycin (0.3 mg·kg^−1^ or 0.6 mg·kg^−1^ thrice weekly), or a combination of 0.3 mithramycin and gemcitabine, or 0.6 mithramycin and gemcitabine. Tumor volume measured at the endpoint (A). Tumors were probed with a cleaved caspase 3 antibody and quantified with Image J. Images were acquired at 20× magnification (B).

*In vivo* subcutaneous injection of MIA PaCa‐2 cells into athymic nude mice were injected with either saline, 0.3 mg·kg^−1^ mithramycin (MTH; SP1 inhibitor), 0.6 mg·kg^−1^ MTH, 50 mg·kg^−1^ gemcitabine (GEM), 0.3 mg·kg^−1^ MTH and GEM, or 0.6 mg·kg^−1^ MTH and GEM. Both combinations of MTH and GEM resulted in less tumor volume (0.3 MTH/GEM 304.0 mm^3^; 0.6 MTH/GEM 307.9 mm^3^) than 0.3 mg·kg^−1^ MTH (365.1 mm^3^), 0.6 mg·kg^−1^ MTH (316.3 mm^3^), or GEM (407.2 mm^3^) (Fig. 6A). Further, cleaved caspase 3 was increased in both 0.3 MTH/GEM (7.10) and 0.6 MTH/GEM (8.54) groups compared to single treatments (0.3 MTH 6.56; 0.6 MTH 5.91; GEM 5.24) and control (4.31) (Fig. 6B). Figure S5 provides histological evidence that mithramycin sufficiently downregulates (A) SP1 and (B) GRP78.

## 4 Discussion

Various stressful conditions such as hypoxia, nutrient deprivation, pH changes, or poor vascularization can be growth limiting for tumor cells and thus activate the UPR (Avril *et al*., 2017; Lee, 2007; Ma and Hendershot, 2004). As the cancer cells undergo rapid proliferation, the need for increased protein and other bimolecular synthesis contributes to an increased ER stress response in cancer cells (Avril *et al*., 2017; Lee, 2007). The ER is the main site for the translation of excess nutrition into metabolic and inflammatory responses. In tumor cells, ER stress may restore homeostasis and make the adjacent environment hospitable for tumor survival and tumor expansion, and thus is considered cytoprotective (Healy *et al*., 2009; Ma and Hendershot, 2004; Martinon, 2012). This makes the ER stress response one of the key survival responses in cancer.

Chemoresistance in cancer has been shown to occur via a number of mechanisms. Some studies suggest that GRP78 membrane localization contributes to pro‐proliferative pathways (Lee, 2014; Roller and Maddalo, 2013). While GRP78 membrane localization is a novel and interesting concept in cancer biology, our study finds that chemoresistance in pancreatic cancer cells can be mediated by an overexpression and increased activity of ABC transporter genes. Our data show that the expression of a number of genes in this superfamily correlates with the expression of GRP78 (Table 1). The ABC transporters typically efflux the chemotherapeutic drugs from the cells, thereby minimizing their accumulation in the cells. These pumps are regulated by the ATP in the cells and are transcriptionally regulated by the transcription factor, NRF2, which binds to antioxidant response element in the promoter gene of these genes leading to their upregulation. Normal substrates for the ABC transporters include glutathione and glucuronide conjugates, which can be mediated through NRF2‐ARE activity (Choudhuri and Klaassen, 2006; Krishna and Mayer, 2000), which suggests that NRF2 and ABC transporters can alternatively work in tandem to promote chemoresistance. Further, Kras mutations have been shown to increase transcription and basal levels of NRF2 in cancer, which minimize intracellular ROS accumulation and maintain cancer cell survival (DeNicola *et al*., 2011).

ATP‐binding cassette transporters have been an attractive target for many years, because of their role in chemoresistance (Donmez *et al*., 2011). A suggested strategy for battling chemoresistance is to decrease the efflux of the ABC transporters (Krishna and Mayer, 2000). Verapamil is a first‐generation MDR modulator, whereas tariquidar and zosuquidar are third‐generation modulators, which have demonstrated fewer pharmacokinetic interactions with anti‐neoplastics (Bisi *et al*., 2015; Krishna and Mayer, 2000). Downregulation of GRP78 results in sensitizing the pancreatic cancer cells to multiple chemotherapeutic agents currently used in pancreatic cancer (Fig. 2). Our results indicated that this was due to a decrease in the activity of the ABC transporters in these cells. As ABC transporters depend on ATP hydrolysis, we estimated the ATPase activity in pancreatic cancer cells following GRP78 inhibition and drug treatment. GRP78 has ATP‐binding sites to help in protein folding. We demonstrate that siGRP78 modestly increases the total cellular levels of ATP and ATPase, which could indicate that this reduction is partly due to less GRP78 utilization of ATP. Additionally, we have shown that siGRP78 decreases cellular viability, which is also an ATP‐driven mechanism. Interestingly, our data also showed that gemcitabine with siGrp78 decreased the amount of ATPase compared to gemcitabine alone, which could indicate that less ATP is being utilized by the cell to drive ABC transporters (Fig. 3D). Similarly, inhibition of SP1 also showed increased sensitivity to gemcitabine by deregulation of ABC transporters (Fig. 5C) and these effects could be observed both *in vitro* (Fig. 5) and *in vivo* (Fig. 6).

There have been many efforts to target the UPR in recent years, including proteasome inhibitors, and inhibitors targeting GRP78, HSP90, PERK, and IRE1alpha (Wang and Kaufman, 2014). GRP78, one of the regulators of the UPR, is an attractive target because it is responsible for maintaining homeostasis in the ER. Recently, a small molecule GRP78 inhibitor called IT‐139 was shown to sensitize chemoresistant PDAC cells to gemcitabine (Gifford *et al*., 2016). Our previously published data shows that GRP78‐mediated ER homeostasis is dependent on SP1 activity and inhibition of SP1 prevents the homeostasis and pushes the UPR to a chronic ER stress phase, leading to cancer cell death (Dauer *et al*., 2017).

## 5 Conclusion

Our current study is clinically relevant, because mithramycin (SP1 inhibitor) is undergoing clinical trials for lung, esophagus, breast, and GI cancers. Interestingly, SP1 and NRF2 have been recently described as nononcogene addiction genes (Hedrick *et al*., 2016; Kitamura *et al*., 2017). Thus, understanding the interaction between multiple stress pathways (Unfolded Protein Response, Oxidative Stress) can contribute to development of better therapeutic targets to ameliorate the therapeutic resistance.

## Conflict of interest

University of Minnesota has a patent for Minnelide, which has been licensed to Minneamrita Therapeutics, LLC. AKS is the co‐founder and the Chief Scientific Officer of this company. SB is a consultant with Minneamrita Therapeutics, LLC, and this relationship is managed by University of Miami. The remaining authors declare no conflict of interest.

## Author contributions

PD, SB, and AS performed conceptualization; PD and SB performed investigation and formal analysis; PD involved in methodology; SB and AS involved in funding acquisition and resource collection; SB and AS involved in project administration; SB and AS supervised the study; VD, SB, and AS validated the study; PD and SB wrote the manuscript; and PD, NSS, VG, AN, SB, and AS reviewed and edited the manuscript.

## Supporting information


**Fig. S1.** Silencing GRP78 combined with chemotherapeutics decreases viability in pancreatic cancer cell lines.
**Fig. S2.** Silencing GRP78 combined with gemcitabine results in more cell death.
**Fig. S3.** Verapamil combined with chemotherapeutic compounds decreases cell viability in pancreatic cancer cells.
**Fig. S4.** SP1 is required for ER homeostasis and affects chemoresistance in pancreatic cancer cells, similarly to GRP78.
**Fig. S5.** Mithramycin treatment decreases SP1 and GRP78 expression *in vivo*.
**Fig. S6.** Evidence of silencing.
