# Supplementary material for: GRP78‐mediated antioxidant response and ABC transporter activity confers chemoresistance to pancreatic cancer cells
Source: Mol Oncol. 2018 Aug 7;12(9):1498–512. doi: 10.1002/1878-0261.12322 (PMC6120253; doi:10.1002/1878-0261.12322)

Supplementary Figure Legends

Supplementary Figure 1: Silencing GRP78 combined with chemotherapeutics decreases viability in pancreatic cancer cell lines. (A) S2-VP10 cells transfected with siGRP78 and treated with 100 nM gemcitabine, 50 nM paclitaxel, or 5 μM 5-fluorouracil for 24 and 48 hours resulted in decreased viability compared to treatment or silencing alone. (B) Su.86.86 cells transfected with siGRP78 and treated with 400 nM gemcitabine, 50 nM paclitaxel, or 5 μM 5-fluorouracil for 24 and 48 hours resulted in decreased viability compared to treatment or silencing alone.

Supplementary Figure 2: Silencing GRP78 combined with gemcitabine results in more cell death. (A) Quantitation of cleaved caspase 3 staining after MIA PaCa-2 cells were transfected with siGRP78 and treated with 400 nM gemcitabine 24 hours. (B). S2-VP10 cells transfected with non-silencing siRNA (NS) (B-I), 100 nM gemcitabine (B-II), siGRP78 (B-III), or siGRP78 + gemcitabine (B-IV) for 24 hours, and stained with cleaved caspase 3 for apoptosis, and the respective quantitation.

Supplementary Figure 3: Verapamil combined with chemotherapeutic compounds decreases cell viability in pancreatic cancer cells. MIA PaCa-2 cells were treated with 5 μM 5-FU and 100 μM verapamil for 24 hours to determine cell viability. S2-VP10 cells were treated with 100 μM verapamil and either 100 nM gemcitabine, 50 nm paclitaxel, or 5 μM 5-FU for 24 hours to determine cell viability.

Supplementary Figure 4: Sp1 is required for ER homeostasis and affects chemoresistance in pancreatic cancer cells, similarly to GRP78. S2-VP10 cells transfected with siSp1 and treated with 100 nM gemcitabine, 50 nM paclitaxel, or 5 μM 5-fluorouracil for 24

and 48 hours resulted in decreased viability compared to treatment or silencing alone (A). (B) Quantitation of cleaved caspase 3 staining after MIA PaCa-2 cells were transfected with siSp1 and treated with 400 nM gemcitabine 24 hours. (C). S2-VP10 cells transfected with non-silencing siRNA (NS) (C-I), 100 nM gemcitabine (C-II), siSp1 (C-III), or siSp1 + gemcitabine (C-IV) for 24 hours, and stained with cleaved caspase 3 for apoptosis, and the respective quantitation.

Supplementary Figure 5: Mithramycin treatment decreases SP1 and GRP78 expression *in vivo*

Immunohistochemistry with SP1 and GRP78 on subcutaneous tumor implantation: (I) Control (II) Gemcitabine 50 mg/kg (III) Mithramycin 0.3 mg/kg (IV) Mithramycin 0.3 mg/kg + Gemcitabine 50 mg/kg (V) Mithramycin 0.6 mg/kg (VI) Mithramycin 0.6 mg/kg + Gemcitabine 50 mg/kg.

Supplementary Figure 6: Evidence of silencing

(A) GRP78 (siG) was silenced for 24 and 48 hours in MIA PaCa-2 and S2-VP10 cells and compared with non-silenced scrambled control (NS). (B) SP1 (siS) was silenced for 24 and 48 hours in MIA PaCa-2 cells and for 24 hours in S2-VP10 cells and compared with NS.

Supplementary Figure 1

A

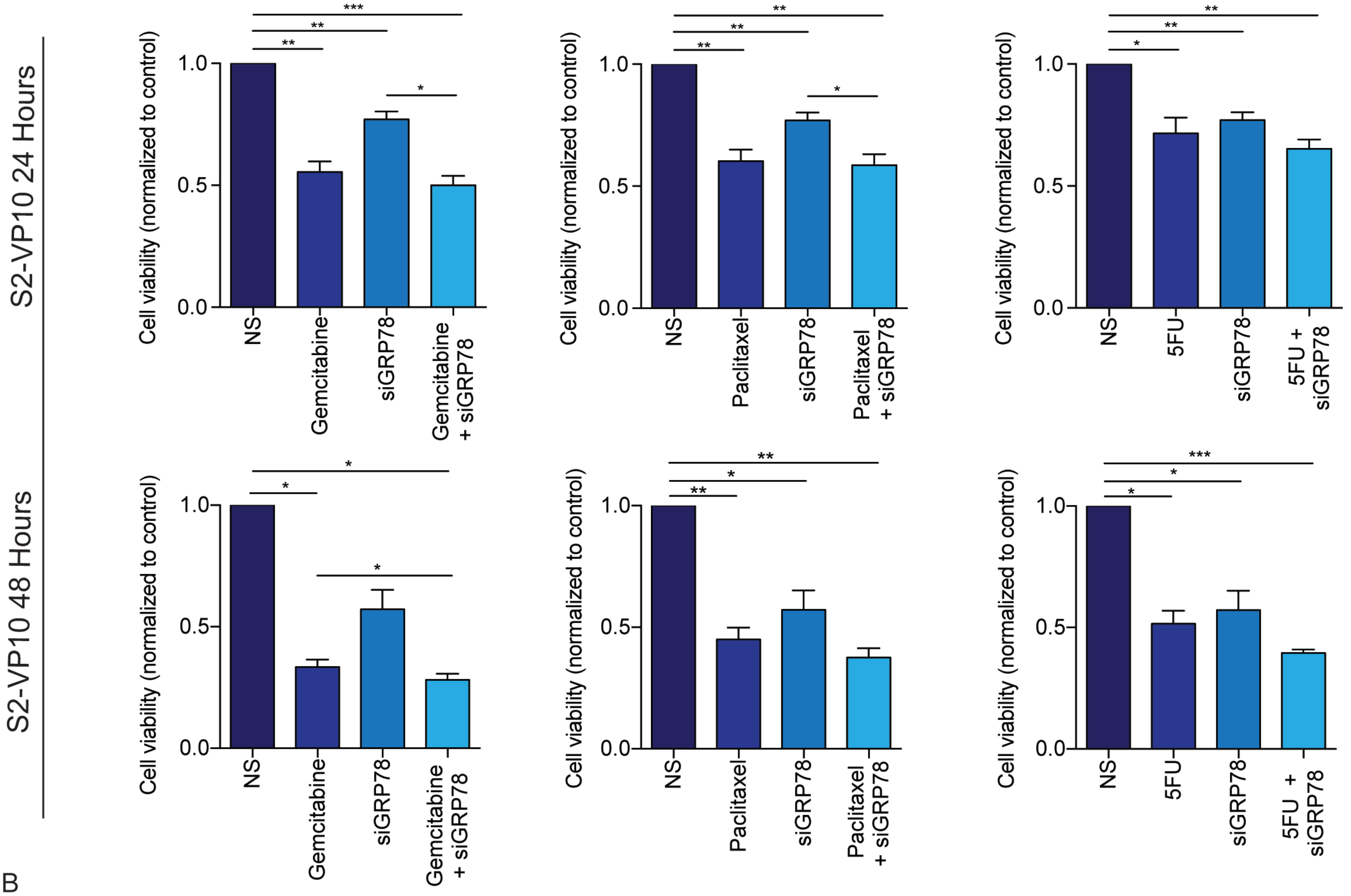

B

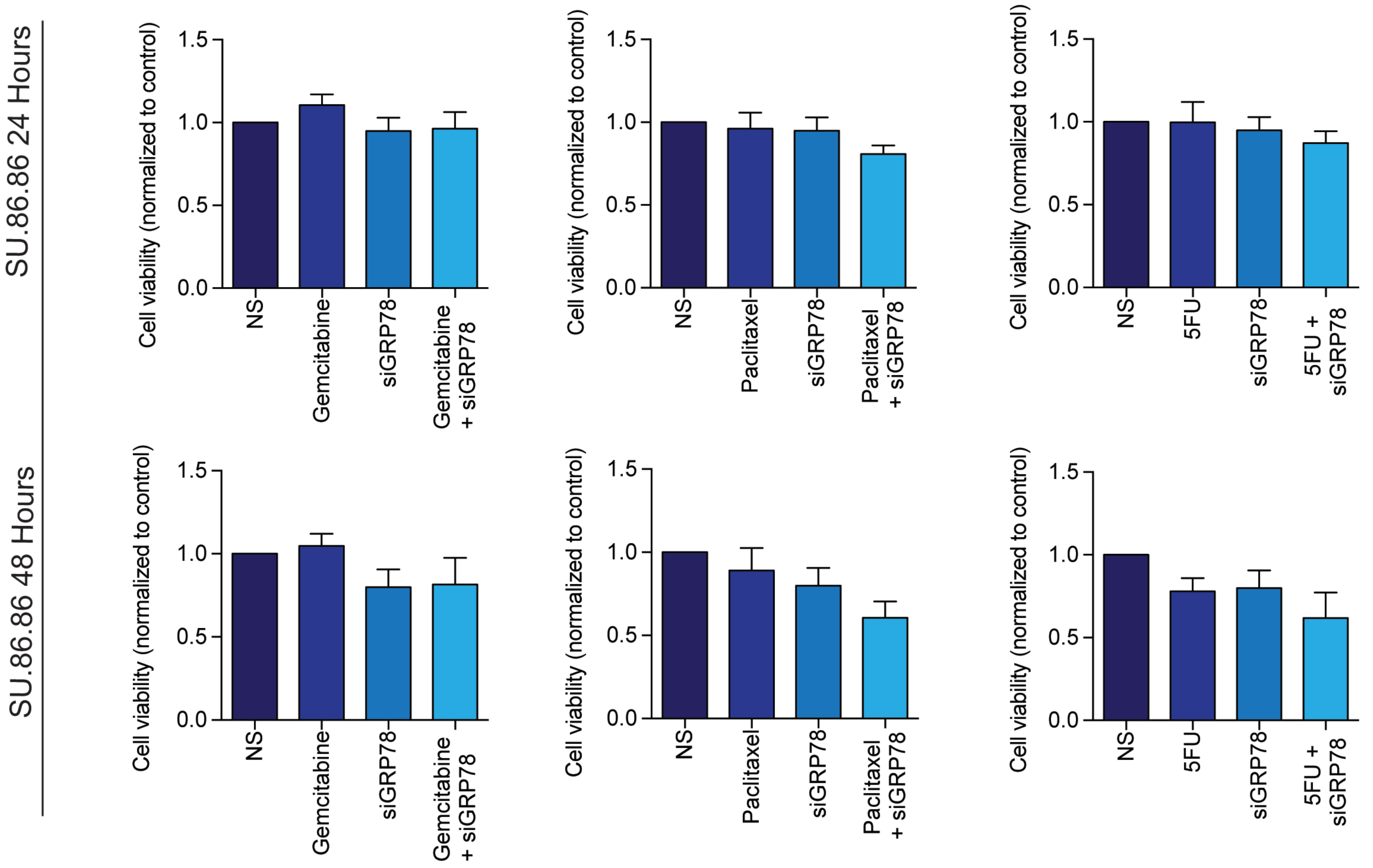

Supplementary Figure 2

A

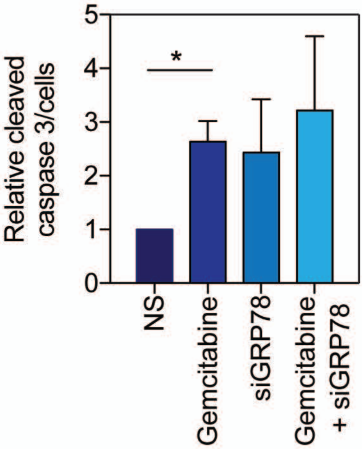

B

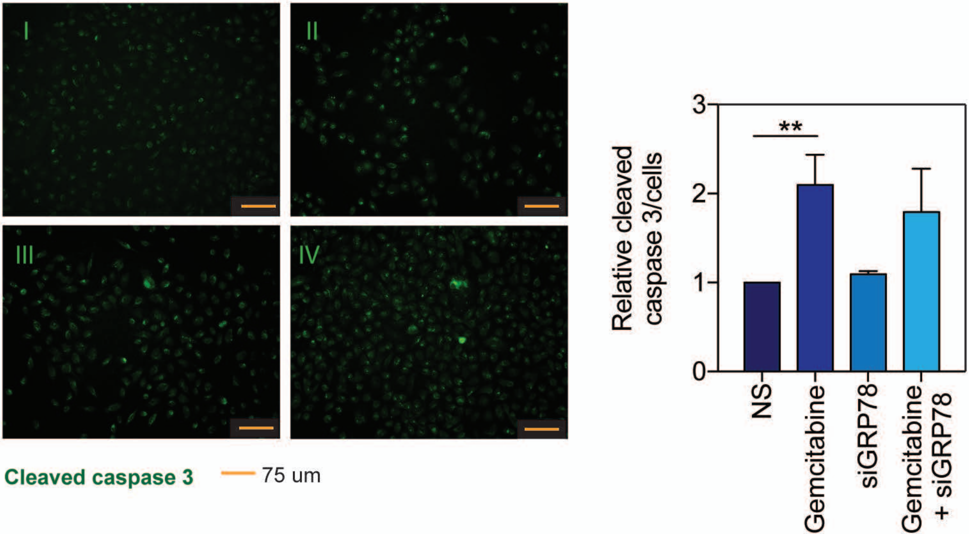

Supplementary Figure 3

A

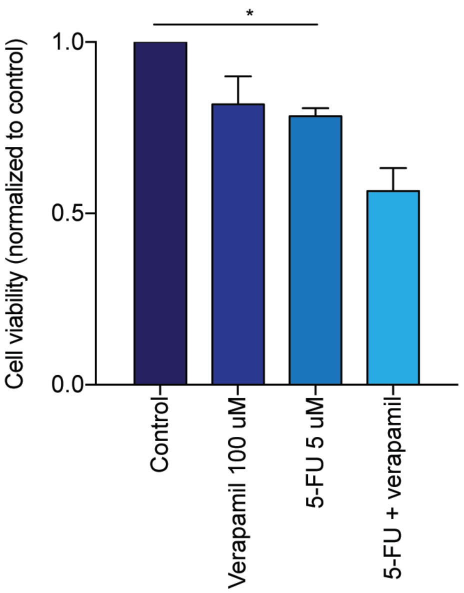

B

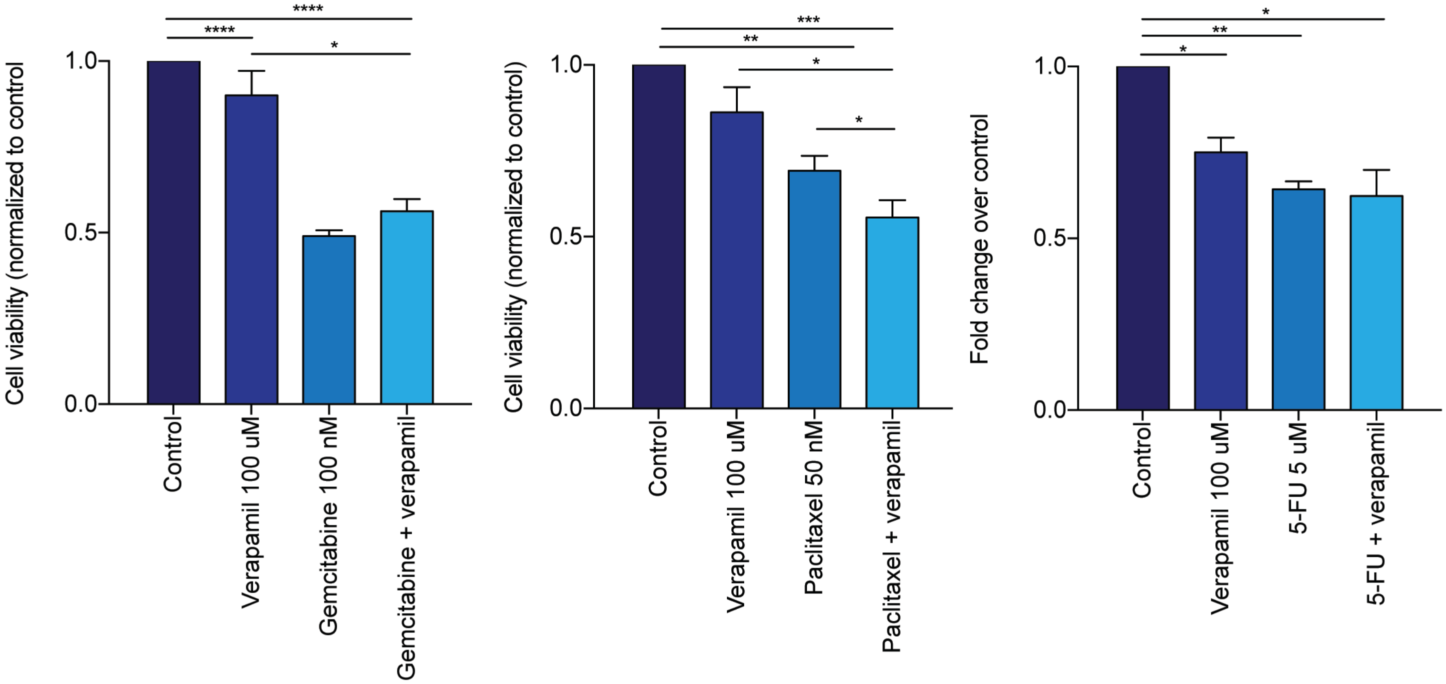

Supplementary Figure 4

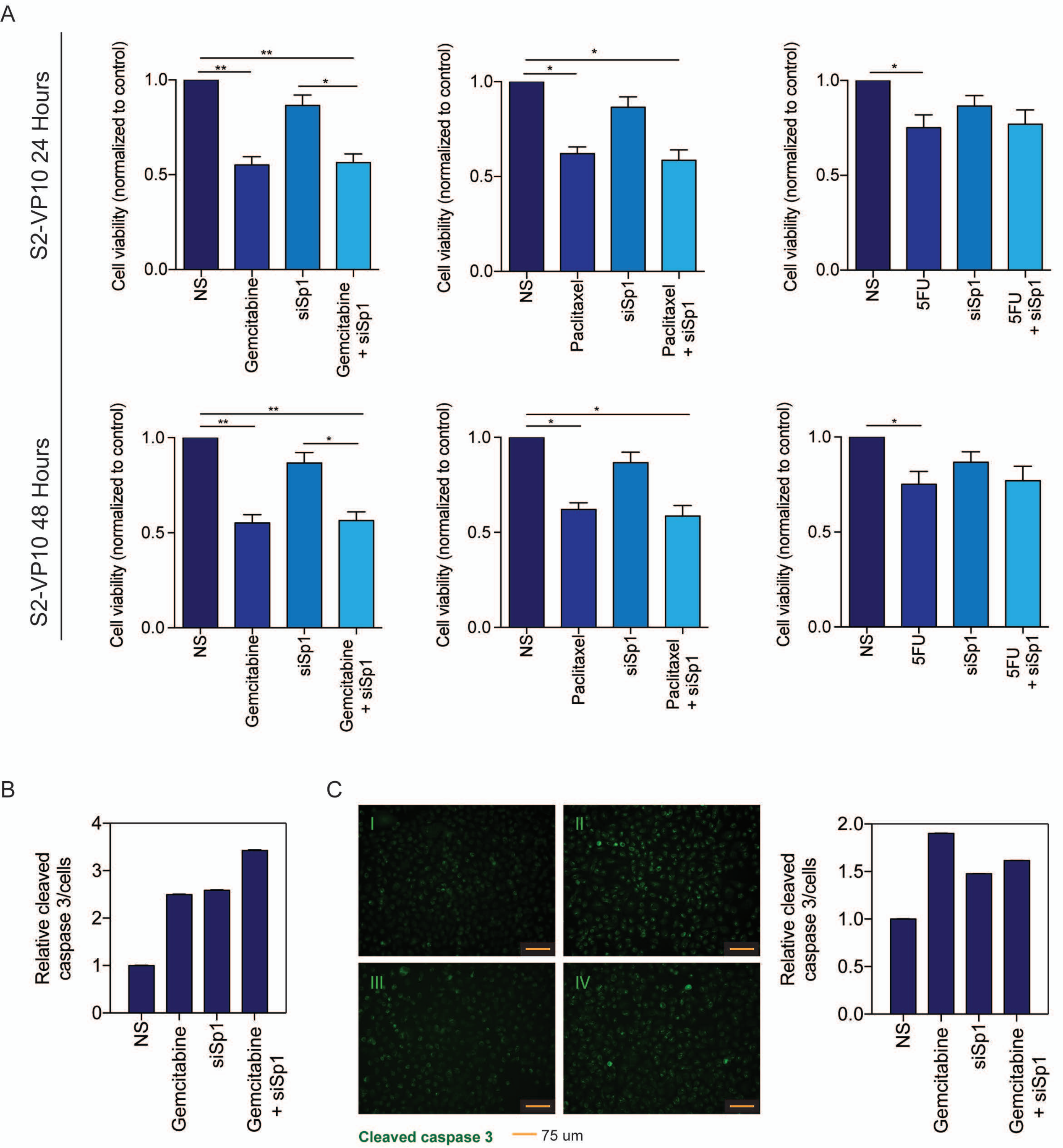

Supplementary Figure 5

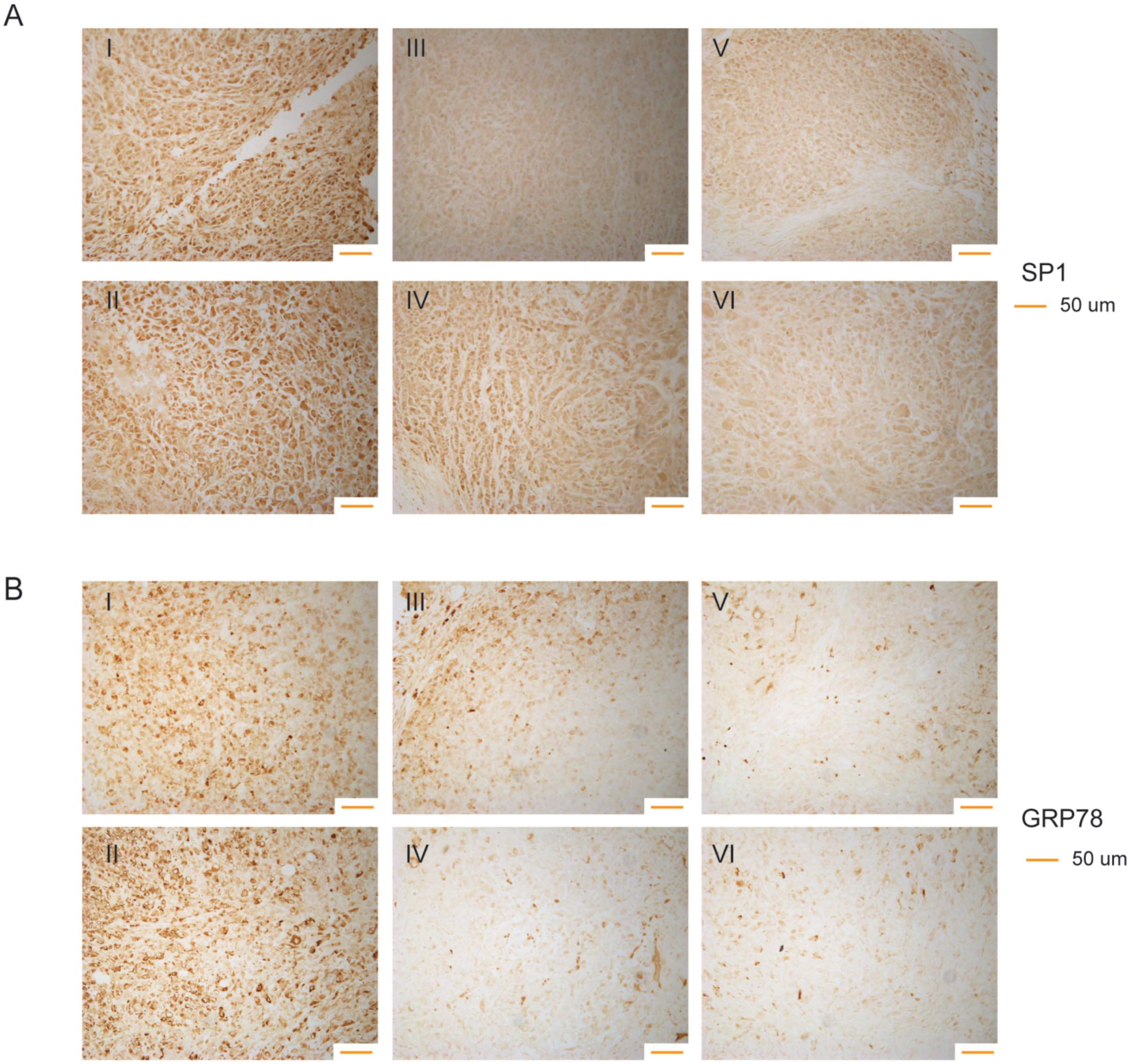

Supplementary Figure 6

A GRP78 silencing

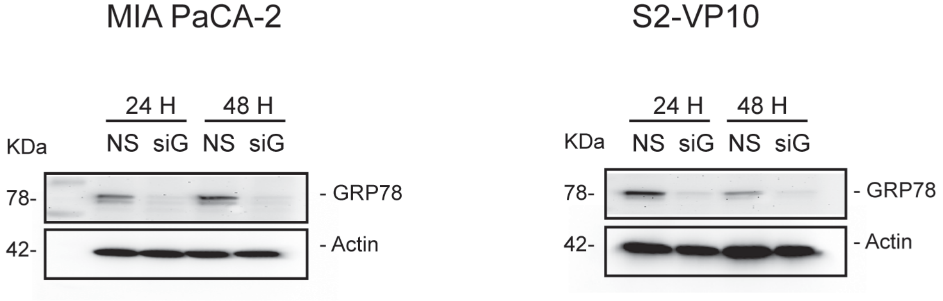

B SP1 silencing

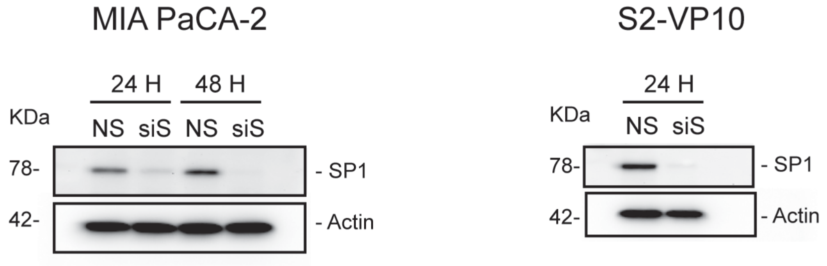

Supplement: Supplementary file 1 — Fig. S1. Silencing GRP78 combined with chemotherapeutics decreases viability in pancreatic cancer cell lines. Fig. S2. Silencing GRP78 combined with gemcitabine results in more cell death. Fig. S3. Verapamil combined with chemotherapeutic compounds decreases cell viability in pancreatic cancer cells. Fig. S4. SP1 is required for ER homeostasis and affects chemoresistance in pancreatic cancer cells, similarly to GRP78. Fig. S5. Mithramycin treatment decreases SP1 and GRP78 expression in vivo. Fig. S6. Evidence of silencing. [file MOL2-12-1498-s001.pdf]
